# Supplementary material for: coupleCoC+: An information-theoretic co-clustering-based transfer learning framework for the integrative analysis of single-cell genomic data
Source: PLoS Comput Biol. 2021 Jun 2;17(6):e1009064. doi: 10.1371/journal.pcbi.1009064 (PMC8202939; doi:10.1371/journal.pcbi.1009064)
Supplement: S1 Text — Text A: coupleCoC+ algorithm. Text B: Summary of coupleCoC+ algorithm. Text C: Selecting Nsub. Text D: Data generation in simulation. (PDF) [file pcbi.1009064.s001.pdf]

Supporting Information - S1 Text

Text A: *couple*CoC+ algorithm

We first reformulate the loss in mutual information into the form of KL divergence [1, 2]. More specifically, for the source data S,

$$\ell_{\mathrm{S}}(C_X, C_Z) = D_{\mathrm{KL}}(p_{\mathrm{S}}(X, Z_{\mathrm{S}})||p^*_{\mathrm{S}}(X, Z_{\mathrm{S}})), \tag{S.1}$$

where $p^*_{\mathrm{S}}(X, Z_{\mathrm{S}})$ is defined as

$$p^*_{\mathrm{S}}(X = x, Z_{\mathrm{S}} = z) = p_{\mathrm{S}}(\tilde{X} = C_X(x), \tilde{Z}_{\mathrm{S}} = C_Z(z)) * \frac{p_{\mathrm{S}}(X = x)}{p_{\mathrm{S}}(\tilde{X} = C_X(x))} \frac{p_{\mathrm{S}}(Z_{\mathrm{S}} = z)}{p_{\mathrm{S}}(\tilde{Z}_{\mathrm{S}} = C_Z(z))}. \tag{S.2}$$

Further, we have

$$\begin{aligned} D_{\mathrm{KL}}(p_{\mathrm{S}}(X, Z_{\mathrm{S}})||p^*_{\mathrm{S}}(X, Z_{\mathrm{S}})) &= \sum_{i=1}^{N_{\mathrm{S}}} \sum_{x \in \{x: C_X(x) = i\}} p_{\mathrm{S}}(X = x) D_{\mathrm{KL}}(p_{\mathrm{S}}(Z_{\mathrm{S}}|X = x)||p^*_{\mathrm{S}}(Z_{\mathrm{S}}|\tilde{X} = i, X = x)) \\ &= \sum_{j=1}^{K} \sum_{z \in \{z: C_Z(z) = j\}} p_{\mathrm{S}}(Z_{\mathrm{S}} = z) D_{\mathrm{KL}}(p_{\mathrm{S}}(X|Z_{\mathrm{S}} = z)||p^*_{\mathrm{S}}(X|\tilde{Z}_{\mathrm{S}} = j, Z_{\mathrm{S}} = z)), \end{aligned} \tag{S.3}$$

where $p^*_{\mathrm{S}}(Z_{\mathrm{S}} = z|\tilde{X} = i, X = x) \triangleq \frac{p^*_{\mathrm{S}}(X=x, Z_{\mathrm{S}}=z)}{p_{\mathrm{S}}(X=x)}$, $z = 1, \ldots, q$, for any $x \in \{x : C_X(x) = i\}$, and $p^*_{\mathrm{S}}(X = x|\tilde{Z}_{\mathrm{S}} = j, Z_{\mathrm{S}} = z) \triangleq \frac{p^*_{\mathrm{S}}(X=x, Z_{\mathrm{S}}=z)}{p_{\mathrm{S}}(Z_{\mathrm{S}}=z)}$, $x = 1, \ldots, n_{\mathrm{S}}$, for any $z \in \{z : C_Z(z) = j\}$. Details on the derivation of formulas (S.1) and (S.3) are presented in [1] and [2]. For the target data T and U, we can rewrite ℓ_{T} and ℓ_{U} similarly as we rewrote ℓ_{S} in formulas (S.1) and (S.3) as follows:

$$\ell_{\mathrm{T}}(C_Y, C_Z) = D_{\mathrm{KL}}(p_{\mathrm{T}}(Y_{\mathrm{T}}, Z_{\mathrm{T}})||p^*_{\mathrm{T}}(Y_{\mathrm{T}}, Z_{\mathrm{T}})), \tag{S.4}$$

and

$$\begin{aligned} D_{\mathrm{KL}}(p_{\mathrm{T}}(Y_{\mathrm{T}}, Z_{\mathrm{T}})||p^*_{\mathrm{T}}(Y_{\mathrm{T}}, Z_{\mathrm{T}})) &= \sum_{i=1}^{N_{\mathrm{T}}} \sum_{y \in \{y: C_Y(y) = i\}} p_{\mathrm{T}}(Y_{\mathrm{T}} = y) D_{\mathrm{KL}}(p_{\mathrm{T}}(Z_{\mathrm{T}}|Y_{\mathrm{T}} = y)||p^*_{\mathrm{T}}(Z_{\mathrm{T}}|\tilde{Y}_{\mathrm{T}} = i, Y_{\mathrm{T}} = y)) \\ &= \sum_{j=1}^{K} \sum_{z \in \{z: C_Z(z) = j\}} p_{\mathrm{T}}(Z_{\mathrm{T}} = z) D_{\mathrm{KL}}(p_{\mathrm{T}}(Y_{\mathrm{T}}|Z_{\mathrm{T}} = z)||p^*_{\mathrm{T}}(Y_{\mathrm{T}}|\tilde{Z}_{\mathrm{T}} = j, Z_{\mathrm{T}} = z)), \end{aligned} \tag{S.5}$$

and

$$\ell_{\mathrm{U}}(C_Y, C_U) = D_{\mathrm{KL}}(p_{\mathrm{U}}(Y_{\mathrm{U}}, Z_{\mathrm{U}})||p^*_{\mathrm{U}}(Y_{\mathrm{U}}, Z_{\mathrm{U}})), \tag{S.6}$$

and

$$\begin{aligned} D_{\mathrm{KL}}(p_{\mathrm{U}}(Y_{\mathrm{U}}, Z_{\mathrm{U}})||p^*_{\mathrm{U}}(Y_{\mathrm{U}}, Z_{\mathrm{U}})) &= \sum_{i=1}^{N_{\mathrm{T}}} \sum_{y \in \{y: C_Y(y) = i\}} p_{\mathrm{U}}(Y_{\mathrm{U}} = y) D_{\mathrm{KL}}(p_{\mathrm{U}}(Z_{\mathrm{U}}|Y_{\mathrm{U}} = y)||p^*_{\mathrm{U}}(Z_{\mathrm{U}}|\tilde{Y}_{\mathrm{U}} = i, Y_{\mathrm{U}} = y)) \\ &= \sum_{j=1}^{K_0} \sum_{u \in \{u: C_U(u) = j\}} p_{\mathrm{U}}(Z_{\mathrm{U}} = u) D_{\mathrm{KL}}(p_{\mathrm{U}}(Y_{\mathrm{U}}|Z_{\mathrm{U}} = u)||p^*_{\mathrm{U}}(Y_{\mathrm{U}}|\tilde{Z}_{\mathrm{U}} = j, Z_{\mathrm{U}} = u)). \end{aligned} \tag{S.7}$$

(Note that K_0 in formula (S.7) denotes the number of feature clusters in data U.) Therefore, the following optimization problem

$$\begin{aligned} \underset{\substack{C_Y, C_X, C_Z, C_U \\ h_{\mathrm{T}, N_{\mathrm{sub}}}, h_{\mathrm{S}, N_{\mathrm{sub}}}}}{\operatorname{argmin}} \quad & \ell_{\mathrm{T}}(C_Y, C_Z) + \lambda \ell_{\mathrm{S}}(C_X, C_Z) + \beta \ell_{\mathrm{U}}(C_Y, C_U) \\ & + \gamma D_{\mathrm{KL}}(\hat{p}_{\mathrm{T}}(\tilde{Y}_{h_{\mathrm{T}, N_{\mathrm{sub}}}}, \tilde{Z}_{\mathrm{T}})||\hat{p}_{\mathrm{S}}(\tilde{X}_{h_{\mathrm{S}, N_{\mathrm{sub}}}}, \tilde{Z}_{\mathrm{S}})). \end{aligned} \tag{S.8}$$

can be rewritten as:

$$\underset{\substack{C_Y,C_X,C_Z,C_U\\ h_{\mathrm{T},N_{\mathrm{sub}}},h_{\mathrm{S},N_{\mathrm{sub}}}}}{\operatorname{argmin}} D_{\mathrm{KL}}(p_{\mathrm{S}}(X,Z_{\mathrm{S}})||p^*_{\mathrm{S}}(X,Z_{\mathrm{S}}))+\lambda D_{\mathrm{KL}}(p_{\mathrm{T}}(Y_{\mathrm{T}},Z_{\mathrm{T}})||p^*_{\mathrm{T}}(Y_{\mathrm{T}},Z_{\mathrm{T}}))+\beta D_{\mathrm{KL}}(p_{\mathrm{U}}(Y_{\mathrm{U}},Z_{\mathrm{U}})||p^*_{\mathrm{U}}(Y_{\mathrm{U}},Z_{\mathrm{U}}))$$
$$+\gamma D_{\mathrm{KL}}(\hat{p}_{\mathrm{T}}(\tilde{Y}_{h_{\mathrm{T},N_{\mathrm{sub}}}},\tilde{Z}_{\mathrm{T}})||\hat{p}_{\mathrm{S}}(\tilde{X}_{h_{\mathrm{S},N_{\mathrm{sub}}}},\tilde{Z}_{\mathrm{S}})) \tag{S.9}$$

Next, the optimization problem (S.9) can be solved by iteratively updating C_Y, C_X, C_Z, C_U, $h_{\mathrm{T},N_{\mathrm{sub}}}$ and $h_{\mathrm{S},N_{\mathrm{sub}}}$ as follows:

- Given C_X, C_Z, C_U, $h_{\mathrm{T},N_{\mathrm{sub}}}$ and $h_{\mathrm{S},N_{\mathrm{sub}}}$, update C_Y. The optimization problem (S.9) is equivalent to minimizing

$$\sum_{i=1}^{N_{\mathrm{T}}}\sum_{y\in\{y:C_Y(y)=i\}} p_{\mathrm{T}}(Y_{\mathrm{T}}=y)Q(\tilde{Y}_{\mathrm{T}}=i,Y_{\mathrm{T}}=y|C_X,C_Z,C_U,h_{\mathrm{S},N_{\mathrm{sub}}},h_{\mathrm{T},N_{\mathrm{sub}}}),$$

where

$$Q(\tilde{Y}_{\mathrm{T}}=i,Y_{\mathrm{T}}=y|C_X,C_Z,C_U,h_{\mathrm{S},N_{\mathrm{sub}}},h_{\mathrm{T},N_{\mathrm{sub}}})\triangleq D_{\mathrm{KL}}(p_{\mathrm{T}}(Z_{\mathrm{T}}|Y_{\mathrm{T}}=y)||p^*_{\mathrm{T}}(Z_{\mathrm{T}}|\tilde{Y}_{\mathrm{T}}=i,Y_{\mathrm{T}}=y))+$$
$$\beta\frac{p_{\mathrm{U}}(Y_{\mathrm{U}}=y)}{p_{\mathrm{T}}(Y_{\mathrm{T}}=y)}D_{\mathrm{KL}}(p_{\mathrm{U}}(Z_{\mathrm{U}}|Y_{\mathrm{U}}=y)||p^*_{\mathrm{U}}(Z_{\mathrm{U}}|\tilde{Y}_{\mathrm{U}}=i,Y_{\mathrm{U}}=y))+\frac{\gamma D_{\mathrm{KL}}(\hat{p}_{\mathrm{T}}(\tilde{Y}_{h_{\mathrm{T},N_{\mathrm{sub}}}},\tilde{Z}_{\mathrm{T}})||\hat{p}_{\mathrm{S}}(\tilde{X}_{h_{\mathrm{S},N_{\mathrm{sub}}}},\tilde{Z}_{\mathrm{S}}))}{n_{\mathrm{T}}p_{\mathrm{T}}(Y=y)}.$$

We iteratively update the cluster assignment $C_Y(y)$ for each cell y ($y=1,\ldots,n_{\mathrm{T}}$) in the target data, fixing the cluster assignment for the other cells:

$$C_Y(y)=\underset{i\in\{1,\ldots,N_{\mathrm{T}}\}}{\operatorname{argmin}} Q(\tilde{Y}_{\mathrm{T}}=i,Y_{\mathrm{T}}=y|C_X,C_Z,C_U,h_{\mathrm{S},N_{\mathrm{sub}}},h_{\mathrm{T},N_{\mathrm{sub}}}). \tag{S.10}$$

- Given C_Y, C_Z, C_U, $h_{\mathrm{T},N_{\mathrm{sub}}}$ and $h_{\mathrm{S},N_{\mathrm{sub}}}$, update C_X. The optimization problem (S.9) is equivalent to minimizing

$$\sum_{j=1}^{N_{\mathrm{S}}}\sum_{x\in\{x:C_X(x)=j\}} p_{\mathrm{S}}(X=x)Q(\tilde{X}=j,X=x|C_Y,C_Z,C_U,h_{\mathrm{S},N_{\mathrm{sub}}},h_{\mathrm{T},N_{\mathrm{sub}}}),$$

where

$$Q(\tilde{X}=j,X=x|C_Y,C_Z,C_U,h_{\mathrm{S},N_{\mathrm{sub}}},h_{\mathrm{T},N_{\mathrm{sub}}})$$
$$\triangleq\lambda D_{\mathrm{KL}}(p_{\mathrm{S}}(Z_{\mathrm{S}}|X=x)||p^*_{\mathrm{S}}(Z_{\mathrm{S}}|\tilde{X}=j,X=x))+\frac{\gamma D_{\mathrm{KL}}(\hat{p}_{\mathrm{T}}(\tilde{Y}_{h_{\mathrm{T},N_{\mathrm{sub}}}},\tilde{Z}_{\mathrm{T}})||\hat{p}_{\mathrm{S}}(\tilde{X}_{h_{\mathrm{S},N_{\mathrm{sub}}}},\tilde{Z}_{\mathrm{S}}))}{n_{\mathrm{S}}p_{\mathrm{S}}(X=x)}.$$

We iteratively update the cluster assignment $C_X(x)$ for each cell x ($x=1,\ldots,n_{\mathrm{S}}$) in the source data, fixing the cluster assignment for the other cells:

$$C_X(x)=\underset{j\in\{1,\ldots,N_{\mathrm{S}}\}}{\operatorname{argmin}} Q(\tilde{X}=j,X=x|C_Y,C_Z,C_U,h_{\mathrm{S},N_{\mathrm{sub}}},h_{\mathrm{T},N_{\mathrm{sub}}}). \tag{S.11}$$

- Given C_X, C_Y, C_U, $h_{\mathrm{T},N_{\mathrm{sub}}}$ and $h_{\mathrm{S},N_{\mathrm{sub}}}$, update C_Z. The optimization problem (S.9) is equivalent to minimizing

$$\sum_{s=1}^{K}\sum_{z\in\{z:\tilde{Z}(z)=s\}} R(\tilde{Z}_{\mathrm{T}}=s,Z_{\mathrm{T}}=z|C_Y,C_X,C_U,h_{\mathrm{S},N_{\mathrm{sub}}},h_{\mathrm{T},N_{\mathrm{sub}}}),$$

where

$$R(\tilde{Z}_{\mathrm{T}}=s,Z_{\mathrm{T}}=z|C_Y,C_X,C_U,h_{\mathrm{S},N_{\mathrm{sub}}},h_{\mathrm{T},N_{\mathrm{sub}}})\triangleq p_{\mathrm{T}}(Z_{\mathrm{T}}=z)D_{\mathrm{KL}}(p_{\mathrm{T}}(Y_{\mathrm{T}}|Z_{\mathrm{T}}=z)||p^*_{\mathrm{T}}(Y_{\mathrm{T}}|\tilde{Z}_{\mathrm{T}}=s,Z_{\mathrm{T}}=z))$$
$$+\lambda p_{\mathrm{S}}(Z_{\mathrm{S}}=z)D_{\mathrm{KL}}(p_{\mathrm{S}}(X|Z_{\mathrm{S}}=z)||p^*_{\mathrm{S}}(X|\tilde{Z}_{\mathrm{S}}=s,Z_{\mathrm{S}}=z))+\frac{\gamma D_{\mathrm{KL}}(\hat{p}_{\mathrm{T}}(\tilde{Y}_{h_{\mathrm{T},N_{\mathrm{sub}}}},\tilde{Z}_{\mathrm{T}})||\hat{p}_{\mathrm{S}}(\tilde{X}_{h_{\mathrm{S},N_{\mathrm{sub}}}},\tilde{Z}_{\mathrm{S}}))}{q}.$$

We iteratively update the cluster assignment $C_Z(z)$ for each feature z ($z = 1, \ldots, q$), fixing the cluster assignment for the other features:

$$C_Z(z) = \underset{s \in \{1,\ldots,K\}}{\operatorname{argmin}} \; R(\tilde{Z}_{\mathrm{T}} = s, Z_{\mathrm{T}} = z | C_Y, C_X, C_U, h_{\mathrm{S},N_{\mathrm{sub}}}, h_{\mathrm{T},N_{\mathrm{sub}}}). \qquad \text{(S.12)}$$

- Given C_Y, update C_U. The optimization problem (S.9) is equivalent to minimizing

$$\sum_{s=1}^{K_0} \sum_{u \in \{u: \tilde{Z}(u)=s\}} p_{\mathrm{U}}(Z_{\mathrm{U}} = u) D_{\mathrm{KL}}(p_{\mathrm{U}}(Y_{\mathrm{U}}|Z_{\mathrm{U}} = u) || p^*_{\mathrm{U}}(Y_{\mathrm{U}}|\tilde{Z}_{\mathrm{U}} = s, Z_{\mathrm{U}} = u)).$$

  We iteratively update the cluster assignment $C_U(u)$ for each feature u ($u = 1, \ldots, q_0$)(Note that q_0 denotes the number of features in data U.), fixing the cluster assignment for the other features:

$$C_U(u) = \underset{s \in \{1,\ldots,K_0\}}{\operatorname{argmin}} \; p_{\mathrm{U}}(Z_{\mathrm{U}} = u) D_{\mathrm{KL}}(p_{\mathrm{U}}(Y_{\mathrm{U}}|Z_{\mathrm{U}} = u) || p^*_{\mathrm{U}}(Y_{\mathrm{U}}|\tilde{Z}_{\mathrm{U}} = s)). \qquad \text{(S.13)}$$

- Given C_X, C_Y, C_Z, C_U, update $h_{\mathrm{T},N_{\mathrm{sub}}}$ and $h_{\mathrm{S},N_{\mathrm{sub}}}$. The optimization problem (S.9) is equivalent to minimizing $D_{\mathrm{KL}}(\hat{p}_{\mathrm{T}}(\tilde{Y}_{h_{\mathrm{T},N_{\mathrm{sub}}}}, \tilde{Z}_{\mathrm{T}}) || \hat{p}_{\mathrm{S}}(\tilde{X}_{h_{\mathrm{S},N_{\mathrm{sub}}}}, \tilde{Z}_{\mathrm{S}}))$. So, we obtain the optimal combinations by

$$(h_{\mathrm{T},N_{\mathrm{sub}}}, h_{\mathrm{S},N_{\mathrm{sub}}}) = \underset{\substack{h_{\mathrm{T},N_{\mathrm{sub}}} \\ h_{\mathrm{S},N_{\mathrm{sub}}}}}{\operatorname{argmin}} D_{\mathrm{KL}}(\hat{p}_{\mathrm{T}}(\tilde{Y}_{h_{\mathrm{T},N_{\mathrm{sub}}}}, \tilde{Z}_{\mathrm{T}}) || \hat{p}_{\mathrm{S}}(\tilde{X}_{h_{\mathrm{S},N_{\mathrm{sub}}}}, \tilde{Z}_{\mathrm{S}})). \qquad \text{(S.14)}$$

  As mentioned in the main text, $h_{\mathrm{T},N_{\mathrm{sub}}}$ is a permutation of size N_{sub} for the indexes of the cell clusters in target data, and there are $\frac{N_{\mathrm{T}}!}{N_{\mathrm{sub}}!(N_{\mathrm{T}} - N_{\mathrm{sub}})!}$ total combinations of $h_{\mathrm{T},N_{\mathrm{sub}}}$. $h_{\mathrm{S},N_{\mathrm{sub}}}$ is an ordered permutation of size N_{sub} for the indexes of the cell clusters in source data, and there are $\frac{N_{\mathrm{S}}!}{(N_{\mathrm{S}} - N_{\mathrm{sub}})!}$ total combinations of $h_{\mathrm{S},N_{\mathrm{sub}}}$. We first calculate the distributions $\tilde{p}_{\mathrm{T}}(\tilde{Y}_{\mathrm{T}}, \tilde{Z}_{\mathrm{T}})$ and $\tilde{p}_{\mathrm{S}}(\tilde{X}, \tilde{Z}_{\mathrm{S}})$ based on C_Y, C_X and Equation (1) in the main text (the expression of $\tilde{p}_{\mathrm{T}}(\tilde{Y}_{\mathrm{T}}, \tilde{Z}_{\mathrm{T}})$ is similar to Equation (1)). The rows in the these two matrices are then normalized so the sum of each row equals to one, which reduces the bias of the differences in the sizes of the clusters in the source data S and the data T. We extract the two submatrices $\tilde{p}_{\mathrm{T}}(\tilde{Y}_{h_{\mathrm{T},N_{\mathrm{sub}}}}, \tilde{Z}_{\mathrm{T}})$ and $\tilde{p}_{\mathrm{S}}(\tilde{X}_{h_{\mathrm{S},N_{\mathrm{sub}}}}, \tilde{Z}_{\mathrm{S}})$. Both can be interpreted as the low dimension representations of the subsets of cell clusters, which are further scaled to have total sums equal to 1, and we then obtain the $\hat{p}_{\mathrm{T}}(\tilde{Y}_{h_{\mathrm{T},N_{\mathrm{sub}}}}, \tilde{Z}_{\mathrm{T}})$ and $\hat{p}_{\mathrm{T}}(\tilde{Y}_{h_{\mathrm{S},N_{\mathrm{sub}}}}, \tilde{Z}_{\mathrm{S}})$. Finally, we calculate the KL divergence between the normalized submatrices $\hat{p}_{\mathrm{T}}(\tilde{Y}_{h_{\mathrm{T},N_{\mathrm{sub}}}}, \tilde{Z}_{\mathrm{T}})$ and $\hat{p}_{\mathrm{S}}(\tilde{X}_{h_{\mathrm{S},N_{\mathrm{sub}}}}, \tilde{Z}_{\mathrm{S}})$.

Text B: Summary of the *couple*CoC+ algorithm

To summarize, the procedures of the *couple*CoC+ algorithm are as follows:

1. Initialization. Calculate $p_{\mathrm{S}}(X, Z_{\mathrm{S}})$, $p_{\mathrm{T}}(Y_{\mathrm{T}}, Z_{\mathrm{T}})$ and $p_{\mathrm{U}}(Y_{\mathrm{U}}, Z_{\mathrm{U}})$ using the datasets S, T and U. Initialize the clustering functions $C_Y^{[0]}$, $C_X^{[0]}$, $C_Z^{[0]}$ and $C_U^{[0]}$. Initialize $p_{\mathrm{T}}^{*[0]}(Y_{\mathrm{T}}, Z_{\mathrm{T}})$ and $p_{\mathrm{S}}^{*[0]}(X, Z_{\mathrm{S}})$. Initialize $h^{[0]}_{\mathrm{T},N_{\mathrm{sub}}}$ and $h^{[0]}_{\mathrm{S},N_{\mathrm{sub}}}$.

2. Iterate steps (a) and (b) until convergence.
   (a). Fix $p_{\mathrm{T}}^{*[t-1]}(Y_{\mathrm{T}}, Z_{\mathrm{T}})$, $p_{\mathrm{S}}^{*[t-1]}(X, Z_{\mathrm{S}})$, $p_{\mathrm{U}}^{*[t-1]}(Y_{\mathrm{U}}, Z_{\mathrm{U}})$, $h^{[t-1]}_{\mathrm{T},N_{\mathrm{sub}}}$ and $h^{[t-1]}_{\mathrm{S},N_{\mathrm{sub}}}$, and sequentially update $C_Y^{[t]}$, $C_X^{[t]}$, $C_Z^{[t]}$, $C_U^{[t]}$, $h^{[t]}_{\mathrm{T},N_{\mathrm{sub}}}$ and $h^{[t]}_{\mathrm{S},N_{\mathrm{sub}}}$ based on the Equations (S.10), (S.11), (S.12), (S.13) and (S.14).
   (b). Fix $C_Y^{[t]}$, $C_X^{[t]}$, $C_Z^{[t]}$, $C_U^{[t]}$, $h^{[t]}_{\mathrm{T},N_{\mathrm{sub}}}$ and $h^{[t]}_{\mathrm{S},N_{\mathrm{sub}}}$, and update $p_{\mathrm{T}}^{*[t]}(Y_{\mathrm{T}}, Z_{\mathrm{T}})$, $p_{\mathrm{S}}^{*[t]}(X, Z_{\mathrm{S}})$ and $p_{\mathrm{U}}^{*[t]}(Y_{\mathrm{U}}, Z_{\mathrm{U}})$.

3. Output the clustering results C_Y, C_X, C_Z, C_U, $h_{\mathrm{T},N_{\mathrm{sub}}}$ and $h_{\mathrm{S},N_{\mathrm{sub}}}$ in the last iteration.

Text C: Selecting $N_{\mathbf{sub}}$

We use the following criteria to choose N_{sub}:

$$g(N_{\mathrm{sub}}) = \frac{\mathrm{Dist}(N_{\mathrm{sub}})}{N_{\mathrm{sub}} * \log(N_{\mathrm{sub}}+1)},$$

where $\mathrm{Dist}(N_{\mathrm{sub}}) \triangleq D_{\mathrm{KL}}(\hat{p}_{\mathrm{T}}(\tilde{Y}_{h_{\mathrm{T},N_{\mathrm{sub}}}}, \tilde{Z}_{\mathrm{T}})||\hat{p}_{\mathrm{S}}(\tilde{X}_{h_{\mathrm{S},N_{\mathrm{sub}}}}, \tilde{Z}_{\mathrm{S}}))$ is obtained when we have C_Y, C_X, C_Z, $h_{\mathrm{T},N_{\mathrm{sub}}}$ and $h_{\mathrm{S},N_{\mathrm{sub}}}$ by solving optimization problem (S.9). There is a bias that KL divergence will tend be larger when N_{sub} is larger, so we penalize it with $N_{\mathrm{sub}} * \log(N_{\mathrm{sub}}+1)$. We choose N_{sub} that has the lowest $g(N_{\mathrm{sub}})$ where $N_{\mathrm{sub}} \in \{1, \ldots, \min\{N_{\mathrm{S}}, N_{\mathrm{T}}\}\}$.

Text D: Data generation in simulation

Similar to the simulation scheme designed by [3], we generate the data T, U and source data S in simulation study as follows (we fix $q = 1000$ and vary w, σ_1, σ_2 and d):

1. Generate $\mathbf{w}^{acc}$ and $\mathbf{w}^{exp}$.

$$w_{rj}^{acc} = \begin{cases} w, & r=1, j=1,\ldots,q(1-w); \\ & r=2, j=q(1-w)+1,\ldots,2q(1-w) \\ 1-w, & r=2, j=1,\ldots,q(1-w); \\ & r=1, j=q(1-w)+1,\ldots,2q(1-w). \end{cases}$$

   $w_{1j}^{acc} = w_{2j}^{acc} \sim Beta(0.5, 2), j = 2q(1-w)+1, \ldots, 2q$. $w_{cj}^{exp} \sim Beta(w_{cj}^{acc}, 10), j = 1, \ldots, 2q(1-w); w_{1j}^{exp} = w_{2j}^{exp} \sim Beta(w_{1j}^{acc}, 10), j = 2q(1-w)+1, \ldots, 2q$.

2. Generate z^{acc} and z^{exp}. The cluster labels are generated with equal probability 0.5.

3. Generate u^{acc} and $\tilde{u}^{acc}$. $\tilde{u}_{ij}^{acc} \sim Bernoulli(0.5)$ if $u_{ij}^{acc} = 1$, where $u_{ij}^{acc} \sim Bernoulli(w_{cj}^{acc})$ if $z_{ic}^{acc} = 1, i = 1, \ldots, n_{\mathrm{S}}; \tilde{u}_{ij}^{acc} = 0$ otherwise.

4. Generate u^{exp} and $\tilde{v}^{exp}$. $\tilde{v}_{lj}^{exp} \sim Bernoulli(0.8)$ if $u_{lj}^{exp} = 1$, where $u_{lj}^{exp} \sim Bernoulli(w_{lj}^{exp})$ if $z_{lc}^{exp} = 1; \tilde{v}_{lj}^{exp} \sim Bernoulli(0.1)$ otherwise; $l = 1, \ldots, n_{\mathrm{T}}$.

5. Generate C and G. $C_{ij} \sim N(0, \sigma_1^2)$ if $\tilde{u}_{ij}^{acc} = 0$ and $C_{ij} \sim N(2, \sigma_1^2)$ if $\tilde{u}_{ij}^{acc} = 1$; $G_{lj} \sim N(0, \sigma_2^2)$ if $\tilde{v}_{lj}^{exp} = 0$ and $G_{lj} \sim N(2, \sigma_2^2)$ if $\tilde{v}_{lj}^{exp} = 1$.

6. Generate T^0 and S^0. $\mathrm{T}_{lj}^0 = 1$ if $\mathrm{G}_{lj} > 0$ and $\mathrm{T}_{lj}^0 = 0$ otherwise; $\mathrm{S}_{ij}^0 = 1$ if $C_{ij} > 0$ and $\mathrm{S}_{ij}^0 = 0$ otherwise.

7. Generate source data S and target data with linked features T. We choose the first q columns of S^0 as the source data S, i.e. $\mathrm{S}_{lj} = \mathrm{S}_{lj}^0, j = 1, \cdots, q$, and choose the first q columns of T^0 as the target data T, i.e. $\mathrm{T}_{lj} = \mathrm{T}_{lj}^0, j = 1, \cdots, q$.

8. Generate target data with unlinked features U by setting $\mathrm{U}_{lj} = \mathrm{T}_{lj}^0 + N(0.5 + I_{\{l=2\}} * d, 0.16), j = q+1, \ldots, 2q$, where d measures the distance between the mean of normal distribution when true label $l = 2$ and the mean of normal distribution when true label $l = 1$.

In our simulation, the difference on the steps of data generation from that in [3] is the addition of Steps 6-8, which generates binary data. $\mathrm{T}^0_{lj} = 1$ means gene j is expressed in cell l, and $\mathrm{T}^0_{lj} = 0$ otherwise. $\mathrm{S}^0_{ij} = 1$ means the promoter region for feature j is accessible in cell i, and $\mathrm{S}^0_{ij} = 0$ otherwise. More details on the notations and the simulation scheme is presented in [3].

References

1. Dhillon IS, Mallela S, Modha DS. Information-theoretic co-clustering. Proceedings of the Ninth ACM SIGKDD International Conference on Knowledge Discovery and Data Mining. 2003; p. 89–98.

2. Dai WY, Yang Q, Xue GR, Yu Y. Self-taught Clustering. Proceedings of the 25th international Conference on Machine Learning. 2008;.

3. Lin ZX, Zamanighomi M, Daley T, Ma S, Wong WH. Model-Based Approach to the Joint Analysis of Single-Cell Data on Chromatin Accessibility and Gene Expression. Stat Sci. 2019;.
