# Supplementary material for: coupleCoC+: An information-theoretic co-clustering-based transfer learning framework for the integrative analysis of single-cell genomic data
Source: PLoS Comput Biol. 2021 Jun 2;17(6):e1009064. doi: 10.1371/journal.pcbi.1009064 (PMC8202939; doi:10.1371/journal.pcbi.1009064)
Supplement: S1 Table — Table A. Clustering table by coupleCoC+ in real data examples 1–4. “clu m” represents the matched cell cluster across the source data and the target data. If there is no “m” in a cell cluster label, it represents that the cluster is not matched across the two datasets, and we use “clu s” and “clu t” to represent that the cluster belongs to source data and target data, respectively. Table B. Enriched functional annotation terms for gene list in the “clu 4” of linked genes in example 1 using DAVID tools. The top 10 terms are shown here. Table C. Enriched functional annotation terms for gene list in the “clu 6” of linked genes in example 1 using DAVID tools. The top 10 terms are shown here. Table D. Summary of the computation time by classical clustering methods SC3 and SIMLR for scRNA-seq data in examples 1–3 and by couple CoC+ for the combination of source data and target data in examples 1–4. The algorithm coupleCoC+ runs until convergence (15 iterations) by MATLAB R2019b—academic use. SC3 and SIMLR run in default iterations in Rstudio (Version 1.2.5033) by the downloaded R packages. All of these algorithms are run in Windows 10 Enterprise (Version 1909) with the Processor: Intel(R) Core(TM)i7–9700 CPU 3.00GHz and with 16.0 GB installed RAM. (PDF) [file pcbi.1009064.s002.pdf]

Supporting Information - S1 Table

Table A. Clustering table by *couple*CoC+ in real data examples 1-4. “clu m” represents the matched cell cluster across the source data and the target data. If there is no “m” in a cell cluster label, it represents that the cluster is not matched across the two datasets, and we use “clu s” and “clu t” to represent that the cluster belongs to source data and target data, respectively.

		*couple*CoC+							
Example 1		clu m1	clu m2	clu m3	clu m4	clu t5	clu t6		
mouse scATAC-seq data (Target data, $n_{\mathrm{T}} = 1525$)	Oligodendrocytes	1	11	446	0	0			
	Astrocytes	529	22	0	0	0			
	Inhibitory neurons	0	110	3	206	0			
	Microglia	0	0	0	0	197			
		clu m1	clu m2	clu m3	clu m4	clu s5	clu s6	clu s7	clu s8
mouse scRNA-seq data (Source data, $n_{\mathrm{S}} = 6539$)	Oligodentrocytes	26	3	62	0	0	0	0	0
	Astrocytes	368	0	0	0	0	0	0	0
	Vip	0	13	0	0	0		1707	8
	Lamp5	0	49	0	0	0	0	28	1045
	Sst	0	153	0	1041	433	114	0	0
	Sncg	0	29	0	0	0	0	91	5
	Serpinf1	0	0	0	0	0	0	19	8
	Pvalb	0	22	0	39	296	980	0	0

		*couple*CoC+		
Example 2		clu m1	clu m2	clu t3
mouse scRNA-seq data (Target data, $n_{\mathrm{T}} = 292$)	pulmonary alveolar type II	0	2	177
	clara	74	23	2
	ependymal	0	14	0
		clu m1	clu m2	clu s3
human scRNA-seq data (Source data, $n_{\mathrm{S}} = 171$)	clara	110	3	
	ependymal	0	58	

		*couple*CoC+	
Example 3		clu m1	clu m2
mouse sc-methylation data (Target data, $n_{\mathrm{T}} = 1102$)	L4	26	386
	L2/3	679	11
		clu m1	clu m2
mouse scRNA-seq data (Source data, $n_{\mathrm{S}} = 2383$)	L4	0	1401
	L2/3 IT	974	8

		*couple*CoC+		
Example 4		clu m1	clu m2	clu t3
batch 2 scRNA-seq data (Target data, $n_{\mathrm{T}} = 288$)	CD141	0	0	96
	pDC	1	88	7
	double negative cells	88	1	7
		clu m1	clu m2	clu s3
batch 1 scRNA-seq data (Source data, $n_{\mathrm{T}} = 288$)	pDC	1	91	4
	double negative cells	89	0	7
	CD1C	2	0	94

Table B. Enriched functional annotation terms for gene list in the "clu 4" of linked genes in example 1 using DAVID tools. The top 10 terms are shown here. The gene list include 59 genes: PLP1, APOD, MOG, PTGDS, TGFA, NDRG1, LIMS2, CNP, MBP, MOBP, MAL, UGT8A, HAPLN2, OPALIN, RNF43, ERMN, PLAT, TNFAIP6, GM15527, PLXNB3, MAG, GJC3, EFEMP1, THSD4, GSN, FA2H, ASPA, NIPAL4, IL12A, LPAR1, GNG11, SEC14L5, BICC1, ENPEP, SPEF2, COBLL1, BCAS1, FOXS1, TSPAN2, PCOLCE, LDLRAP1, SERPINB1A, ASGR1, DDC, GAL3ST1, ST18, HEG1, CNTN2, SHROOM1, GGT6, CCDC121, GJB1, FHDC1, GM7854, INSC, SEPT4, SH3TC2, MCAM, GRB14.

Category	Term	Count	%	Bonferroni P-value
GOTERM_CC_DIRECT	myelin sheath	13	22.03	1.44E-11
GOTERM_BP_DIRECT	myelination	8	13.56	1.12E-07
GOTERM_MF_DIRECT	structural constituent of myelin sheath	4	6.78	2.51E-05
UP_KEYWORDS	Glycoprotein	24	40.68	0.00179
UP_KEYWORDS	Disulfide bond	21	35.59	0.00358
GOTERM_BP_DIRECT	peripheral nervous system myelin maintenance	3	5.08	0.0732
UP_SEQ_FEATURE	glycosylation site:N-linked (GlcNAc...)	23	38.98	0.0501
UP_SEQ_FEATURE	topological domain:Extracellular	16	27.12	0.302
GOTERM_CC_DIRECT	extracellular exosome	17	28.81	0.172
UP_SEQ_FEATURE	lipid moiety-binding region:S-palmitoyl cysteine	5	8.47	0.337

Table C. Enriched functional annotation terms for gene list in the "clu 6" of linked genes in example 1 using DAVID tools. The top 10 terms are shown here. The gene list include 198 genes: HCK, CXCR3, GCNT1, SLA, A630033H20RIK, TMEM173, UPK1B, P2RX7, BC035044, ITPR3, IRGM2, HMHA1, PTPN6, PIK3R5, FCGR1, RASAL3, HPGDS, HAVCR2, H2-Q7, CASP8, SAMSN1, MS4A6B, CD14, ITGAM, HCLS1, LST1, RENBP, TRAF3IP3, GPR183, SLC7A7, ALOX5, EPHA2, WHRN, IRF1, CD33, MYO1F, ENG, I830077J02RIK, CLEC4A3, RHOBTB1, FLT3, BCL2A1B, DUSP27, P2RY6, TNFRSF14, BATF, SLC40A1, FGD2, MFNG, TEC, DOCK8, LYN, HPGD, MRC1, CCL2, HLX, RPS6KA1, KLHL6, 1810011H11RIK, HVCN1, CCL9, CTSC, ANGPTL7, LAIR1, CCDC63, SUSD3, SLFN8, RHBDF2, TGFB1, IL13RA1, CEACAM1, RNASE4, AIF1, POU2F2, EDN1, GBP7, ART3, ADRB2, C430049B03RIK, CYSLTR1, CCL4, H2-T23, IGTP, PLIN2, CD300A, LRRK1, CD68, SLC11A1, CLEC4A2, C3AR1, GNA15, LRP5, CD37, VWF, H2-OA, NFAM1, CD52, RGS1, CSF3R, FLI1, PTPRC, CD86, CORT, LRMP, ST3GAL6, TLR7, LGALS9, IRS3, STAB1, GBP2, GIMAP5, TRIM47, GM12250, CD84, UGT1A7C, CCL3, TNFAIP8L2, NCF4, H2-Q6, LTC4S, CD48, SRGN, LY86, HHEX, PLD4, ALDH1A2, PSMB8, P2RY13, GLIPR1, IL10RA, VAV1, ECM1, BIN2, UCP2, PRKCH, CCL6, GGT5, PRDM1, GIMAP9, LPCAT2, GRAP, NCF1, MSN, ALOX8, LAPTM5, CX3CR1, GPR34, ABCA9, CCR5, KDR, ITGB2, FCGR2B, FILIP1L, GGTA1, KLF2, IFIT1, TMEM119, ECSCR, CSF1R, DLL4, P2RY12, FCRLS, CTSH, CD53, SLFN5, AIM2, TREM2, SLC39A8, FCGR3, XKRX, PECAM1, FAM114A1, ZC3HAV1, CFH, F11R, CTSS, BC028528, GBP9, ARHGDIB, SIGLECH, C1QA, TYROBP, SELPLG, SP100, ACVRL1, GIMAP6, FLT1, TAGLN2, SERPINF1, C1QC, ANXA3, C1QB, FCER1G, CACNA1S, IRF5, H2-EB1, PYCARD.

Category	Term	Count	%	Bonferroni P-value
UP_KEYWORDS	Immunity	35	17.68	8.27E-22
GOTERM_BP_DIRECT	immune system process	34	17.17	3.00E-19
UP_KEYWORDS	Disulfide bond	81	40.91	5.02E-19
GOTERM_BP_DIRECT	inflammatory response	29	14.65	2.81E-15
UP_SEQ_FEATURE	topological domain:Extracellular	69	34.85	2.15E-15
UP_KEYWORDS	Glycoprotein	84	42.42	1.79E-15
UP_KEYWORDS	Innate immunity	23	11.62	2.10E-14
UP_SEQ_FEATURE	topological domain:Cytoplasmic	75	37.88	3.54E-13
UP_SEQ_FEATURE	disulfide bond	69	34.85	7.79E-13
GOTERM_CC_DIRECT	membrane	121	61.11	1.72E-12

Table D. Summary of the computation time by classical clustering methods SC3 and SIMLR for scRNA-seq data in examples 1-3 and by *couple*CoC+ for the combination of source data and target data in examples 1-4. The algorithm *couple*CoC+ runs until convergence (15 iterations) by MATLAB R2019b - academic use. SC3 and SIMLR run in default iterations in Rstudio (Version 1.2.5033) by the downloaded R packages. All of these algorithms are run in Windows 10 Enterprise (Version 1909) with the Processor: Intel(R) Core(TM)i7-9700 CPU 3.00GHz and with 16.0 GB installed RAM.

Examples	Clustering methods		
	SC3	SIMLR	*couple*CoC+ (S+T+U)
Example 1 ($n_{\mathrm{S}} + n_{\mathrm{T}} = 8064$)	20.52(mins)	55.50(mins)	28.20(mins)
Example 2 ($n_{\mathrm{S}} + n_{\mathrm{T}} = 463$)	62.99(s)	32.35(s)	27.82(s)
Example 3 ($n_{\mathrm{S}} + n_{\mathrm{T}} = 3485$)	2.31(mins)	35.15(mins)	7.98(mins)
Example 4 ($n_{\mathrm{S}} + n_{\mathrm{T}} = 576$)	-	-	49.72(s)
