# Supplementary figures and images for: coupleCoC+: An information-theoretic co-clustering-based transfer learning framework for the integrative analysis of single-cell genomic data
Source: PLoS Comput Biol. 2021 Jun 2;17(6):e1009064. doi: 10.1371/journal.pcbi.1009064 (PMC8202939; doi:10.1371/journal.pcbi.1009064)

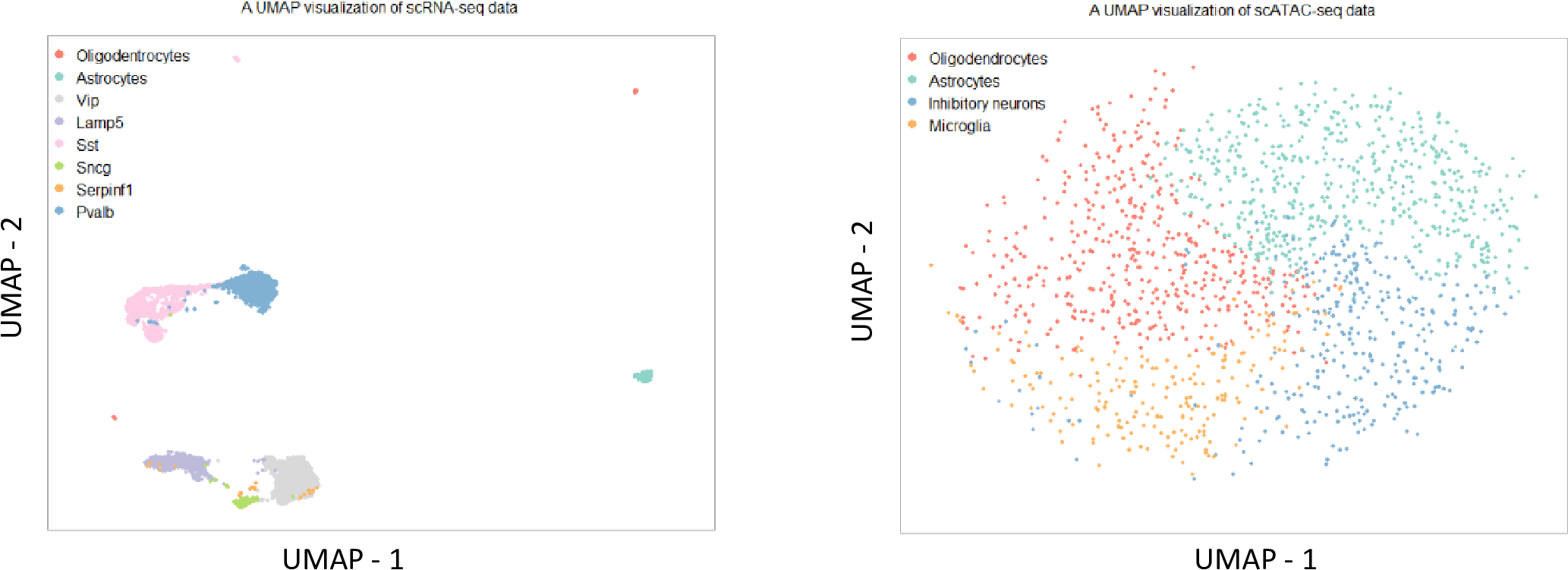

Supplement: S1 Fig — (TIF) [file pcbi.1009064.s003.tif]

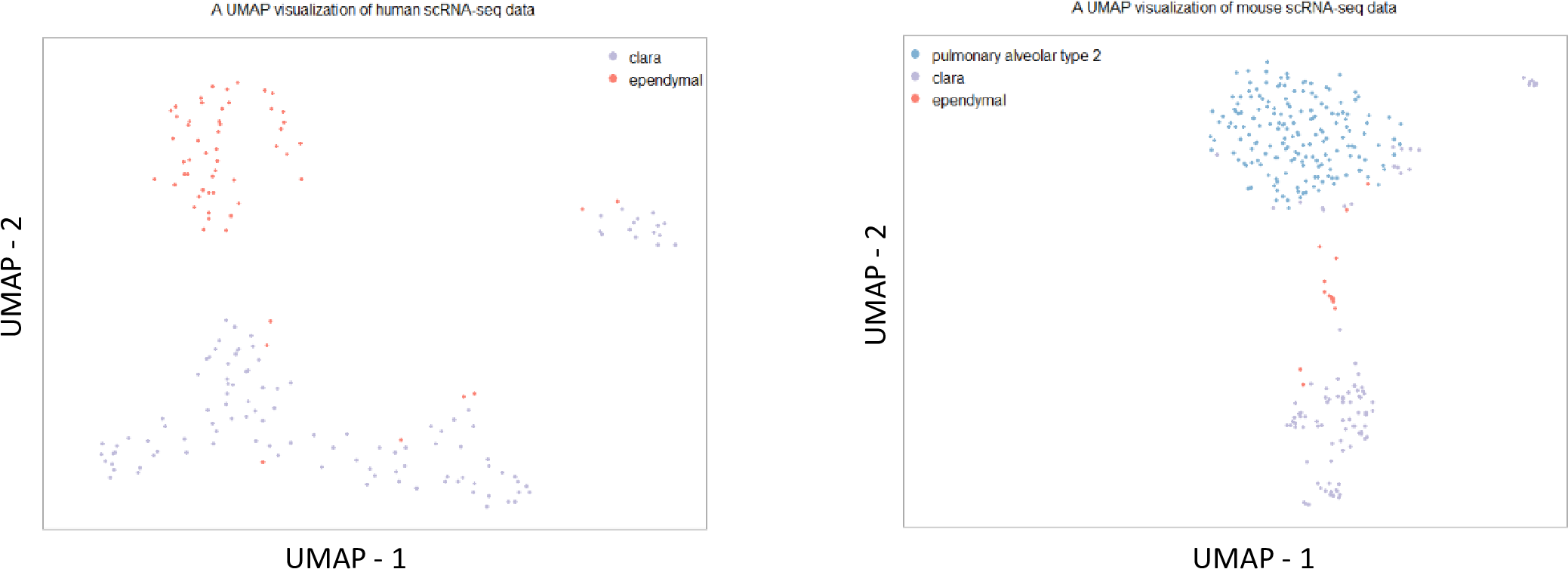

Supplement: S2 Fig — (TIF) [file pcbi.1009064.s004.tif]

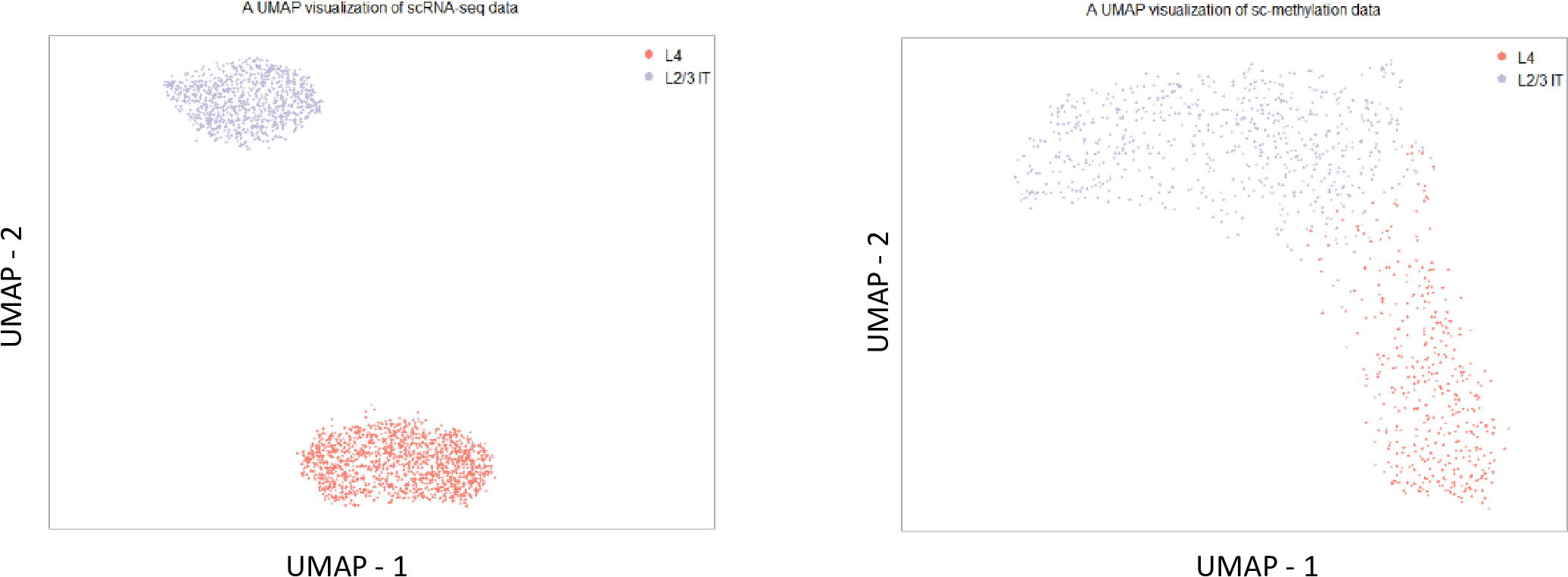

Supplement: S3 Fig — (TIF) [file pcbi.1009064.s005.tif]

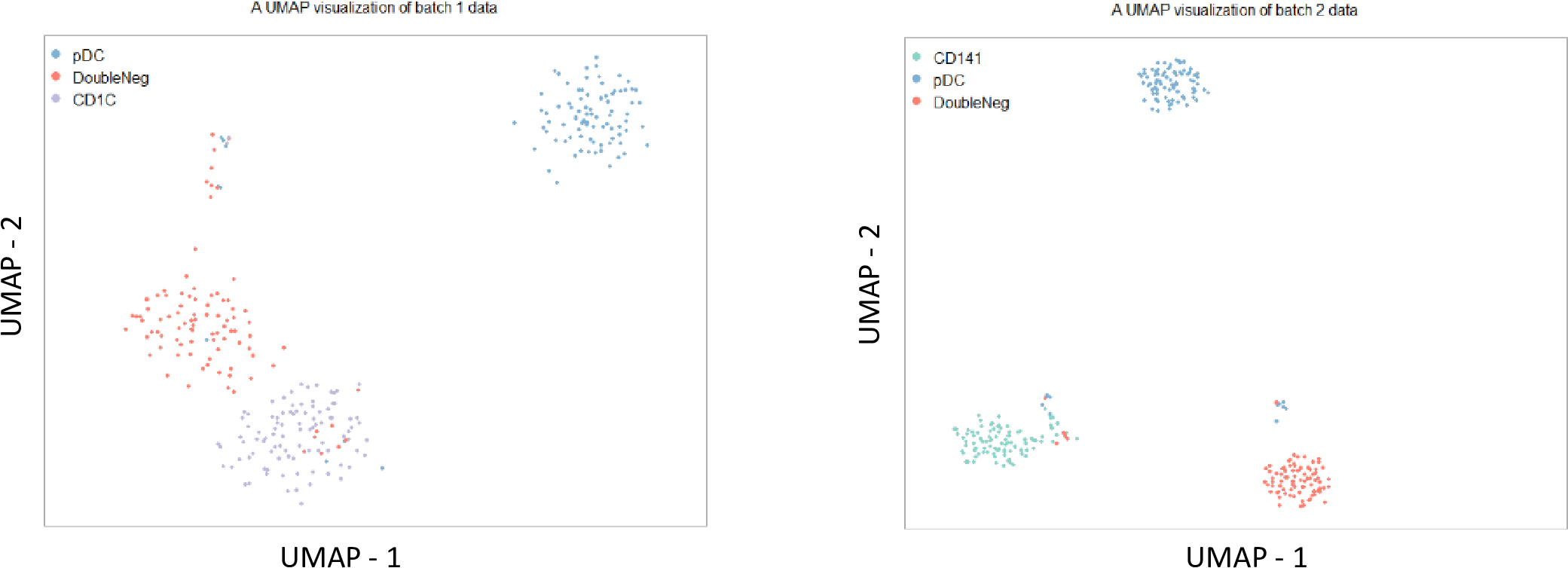

Supplement: S4 Fig — (TIF) [file pcbi.1009064.s006.tif]

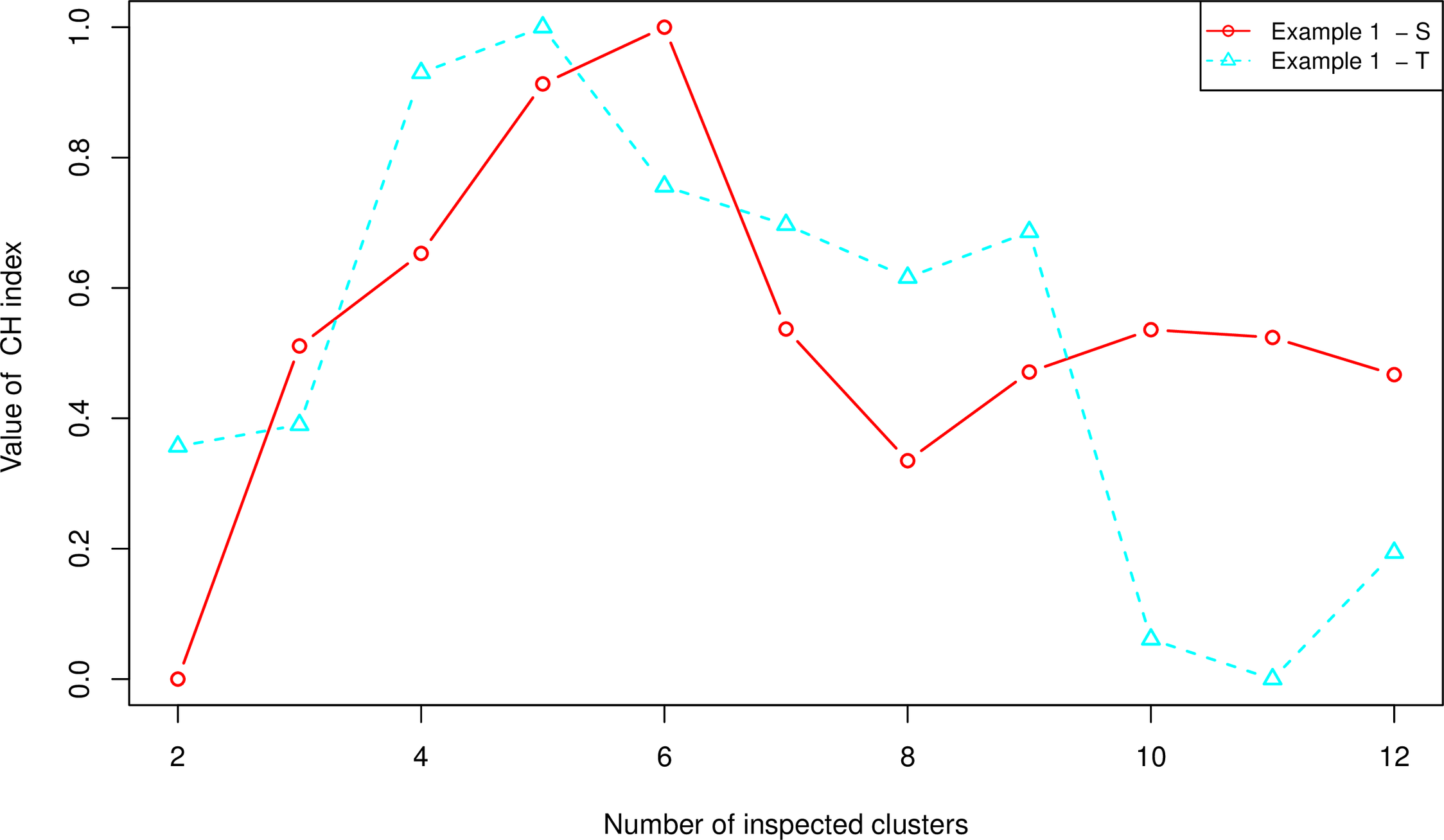

Supplement: S5 Fig — The value of CH index has been standardized via minimax normalization to ensure each value being bound to between 0 and 1. (TIF) [file pcbi.1009064.s007.tif]

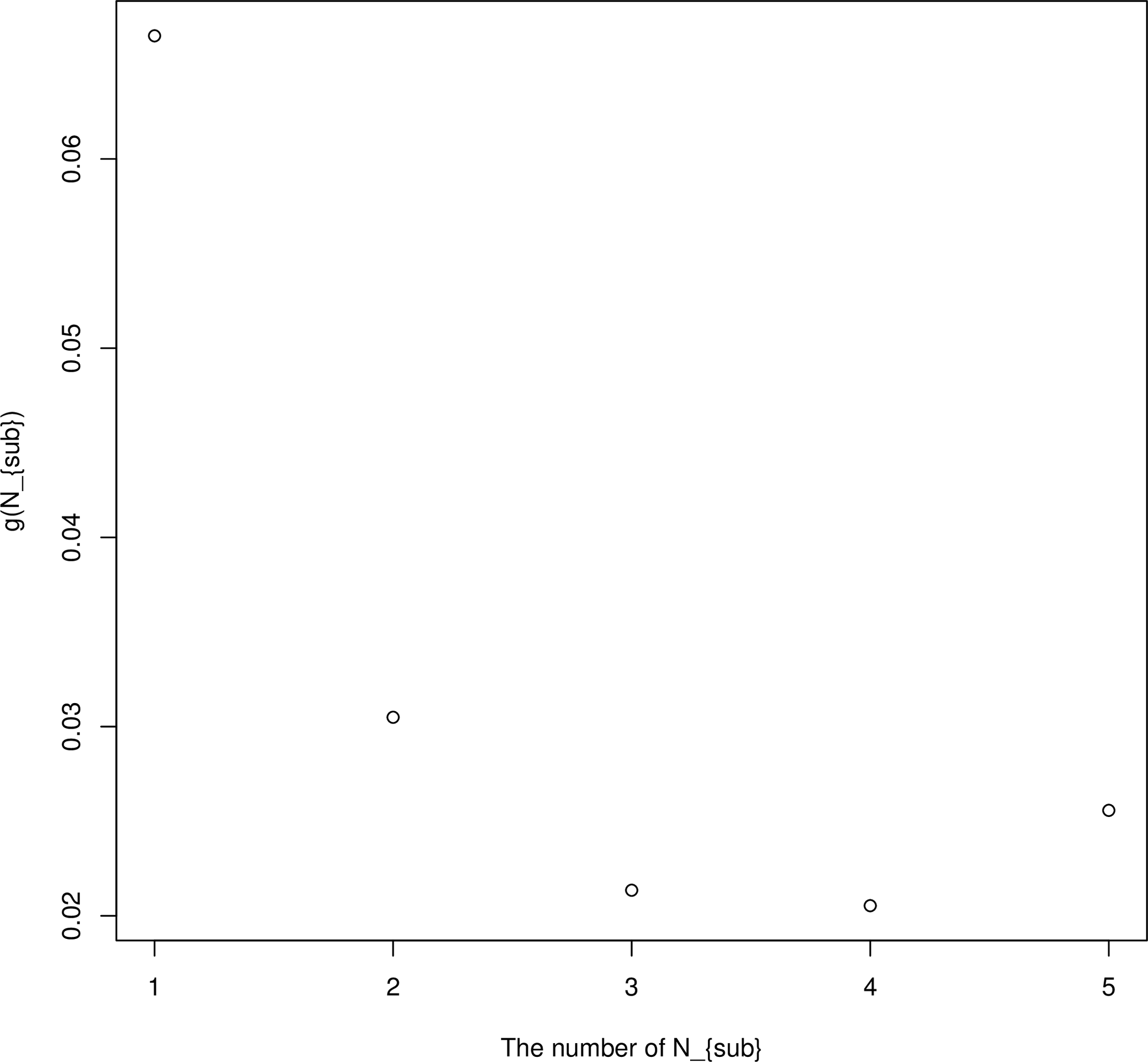

Supplement: S6 Fig — (TIF) [file pcbi.1009064.s008.tif]

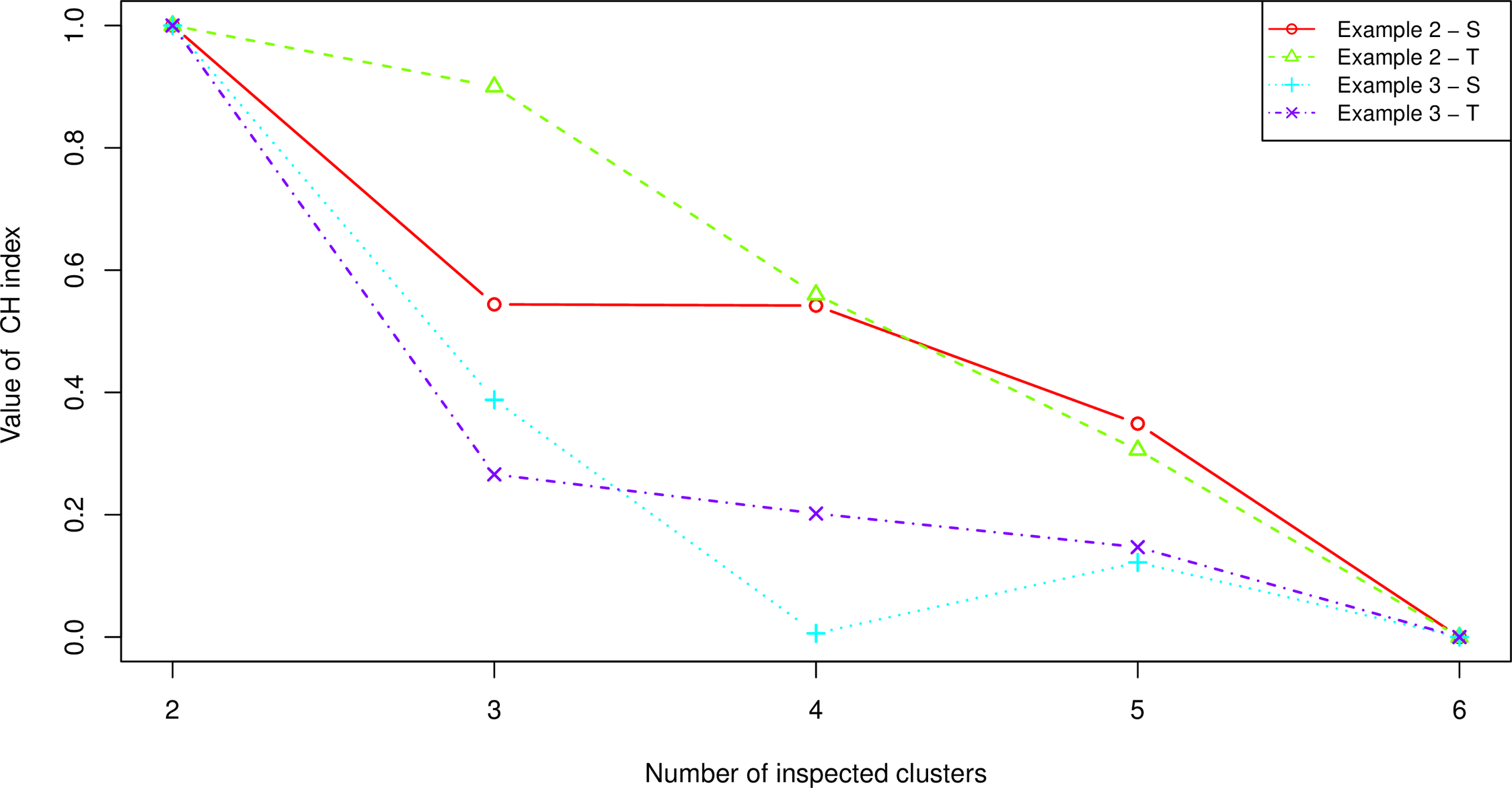

Supplement: S7 Fig — The value of CH index has been standardized via minimax normalization to ensure each value being bound to between 0 and 1. (TIF) [file pcbi.1009064.s009.tif]

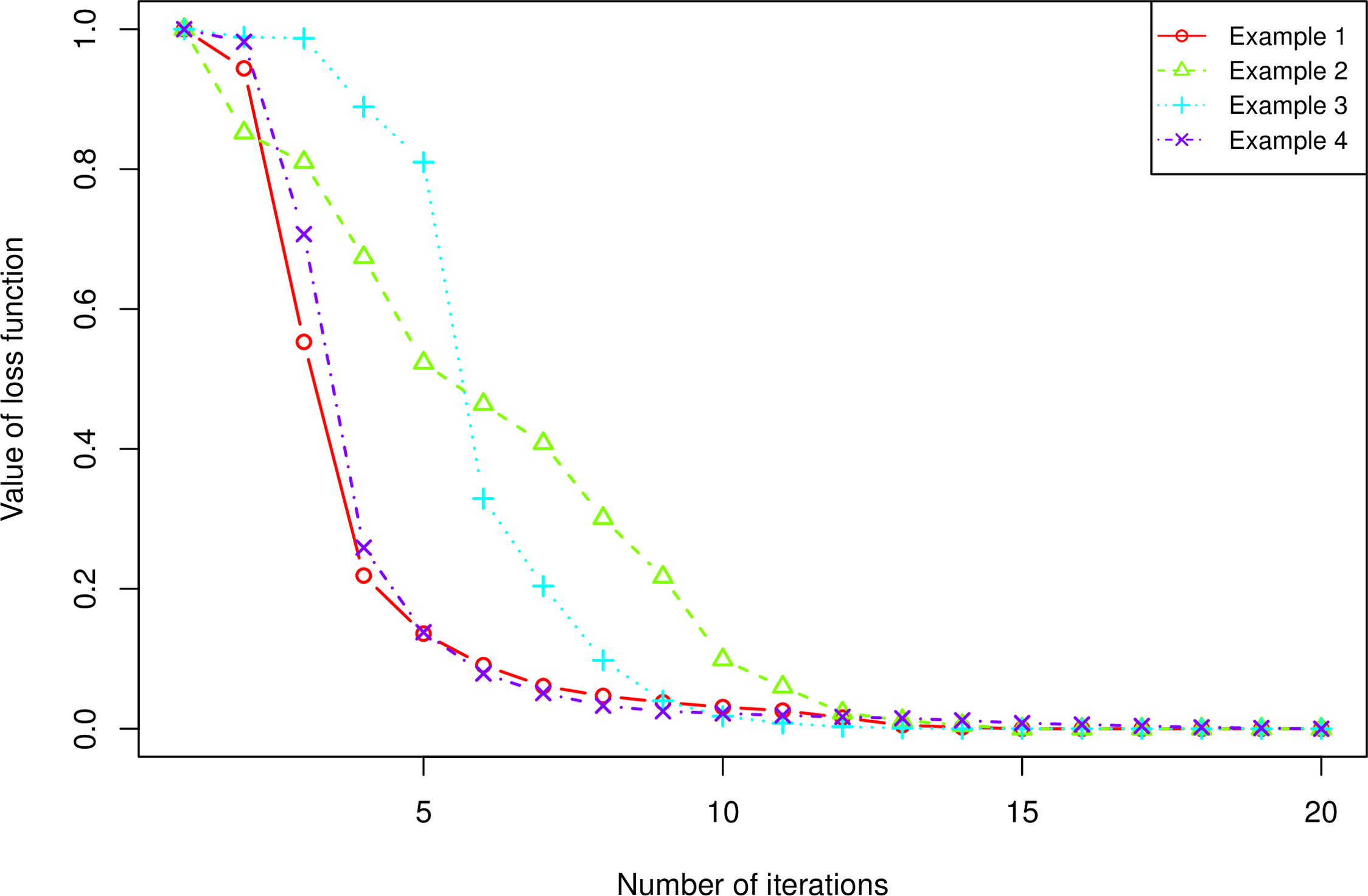

Supplement: S8 Fig — The value of the objective function after each iteration has been standardized via minimax normalization to ensure each value being bound to between 0 and 1. (TIF) [file pcbi.1009064.s010.tif]
