# Supplementary material for: coupleCoC+: An information-theoretic co-clustering-based transfer learning framework for the integrative analysis of single-cell genomic data
Source: PLoS Comput Biol. 2021 Jun 2;17(6):e1009064. doi: 10.1371/journal.pcbi.1009064 (PMC8202939; doi:10.1371/journal.pcbi.1009064)
Supplement: Attachment — Submitted filename: Response to reviewers.pdf [file pcbi.1009064.s011.pdf]

Response to Reviewers for "*couple*CoC+: an information-theoretic co-clustering-based transfer learning framework for the integrative analysis of single-cell genomic data"

Pengcheng Zeng[1] and Zhixiang Lin *[1]

[1]Department of Statistics, The Chinese University of Hong Kong, HK

April 19, 2021

To the reviewers,

We would like to thank you all for your helpful suggestions and constructive comments on our manuscript "*couple*CoC+: an information-theoretic co-clustering-based transfer learning framework for the integrative analysis of single-cell genomic data". Other than the minor changes, we made the following major changes in the package and in the revised manuscript:

1. **We improved our package at github (`https://github.com/cuhklinlab/coupleCoC_plus`) by adding the datasets used in the manuscript, a vignette with examples and the source code for implementing all the benchmark methods.**

2. **We added UMAP visualizations for all real data examples.**

3. **We added biological interpretations to the clustering results in example 1.**

4. **We added a fourth real data example and examined *couple*CoC+ by integrative clustering of human blood dendritic cell (DC) scRNA-seq data from two batches.**

5. **We added simulation studies with 6 settings to examine the performance of *couple*CoC+.**

Next we will clarify the comments raised by the reviewers point by point.

*Corresponding author: zhixianglin@cuhk.edu.hk

Reviewer #1

Zeng and Lin developed *couple*CoC+, a method for integrative analysis of single-cell genomic data. My comments are below.

1. The package only contains matlab scripts with minimal descriptions. Vignettes with toy examples are needed for this to be suited for publication in PLOS Computational Biology.

Response:

Thank you for your constructive comment.

We have improved our package at github `https://github.com/cuhklinlab/coupleCoC_plus` **by adding descriptions about the package in the file "README.md" and adding a vignette with a few examples in the file "vignettes/vignette.md". In the vignette, we included step-by-step analysis for UMAP visualization of raw data, implementation of *couple*CoC+ and the benchmark methods, heatmap visualization and simulation analysis in our manuscript.**

2. For better reproducibility, the scripts used for other methods in the benchmark also need to be uploaded. Also, some visualizations are needed for the three empirical datasets, in addition to the ARI and NMI metrics. Also, how are the metrics calculated, using what ground truths?

Response:

Thank you for your constructive comment.

For better reproducibility, we added the source code for implementing the benchmark methods in the file "vignette.md"(`https://github.com/cuhklinlab/coupleCoC_plus/blob/main/vignettes/vignette.md`**). We also uploaded the data for real examples 1-4, and the simulated data in** `https://github.com/cuhklinlab/coupleCoC_plus/tree/main/data`**.**

Figures R.1-R.4 are the UMAP visualization for all the real datasets, and we added them in the supporting information file *S2 Supplementary Figures* (Fig. S.1.-S.4.)

In this work, we regard the cell type labels provided by the authors in their original papers as the ground truth, which are mostly based on known marker genes. We then calculated the ARI and NMI by using the cell types labels and the cluster assignments given by all the benchmark methods. We added the clarification in the *Results* section in the revised manuscript.

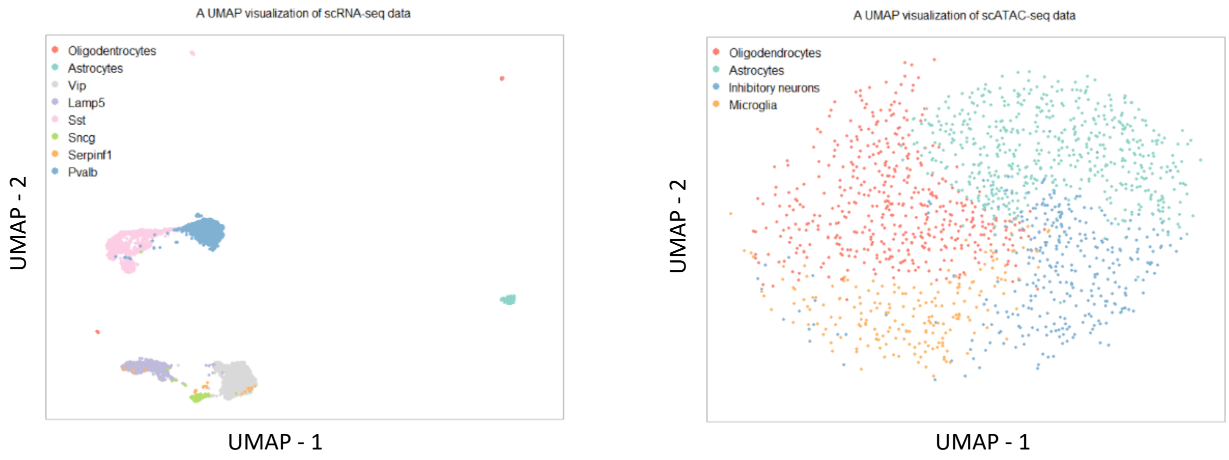

Figure R.1: UMAP visualization of source data (left) and target data (right) in example 1.

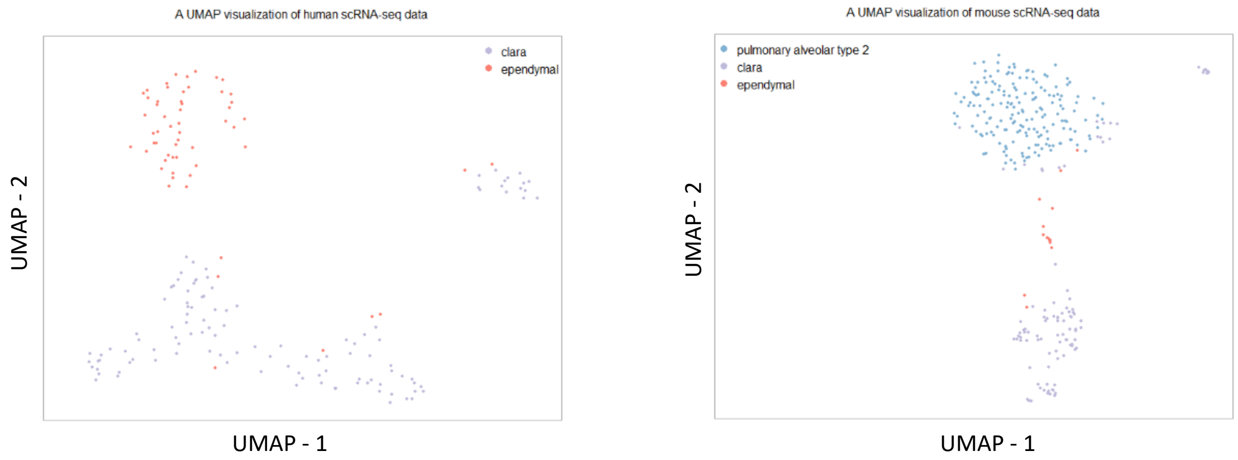

Figure R.2: UMAP visualization of source data (left) and target data (right) in example 2.

3. If one cluster is better separated by one modality, but not the other. How is this handled?

Response:

Thank you for your comment.

If one modality better separates the cell types, the knowledge is transferred to the other modality mainly through them sharing the same feature clusters among the linked features: similar features will be clustered and aggregated from the guidance

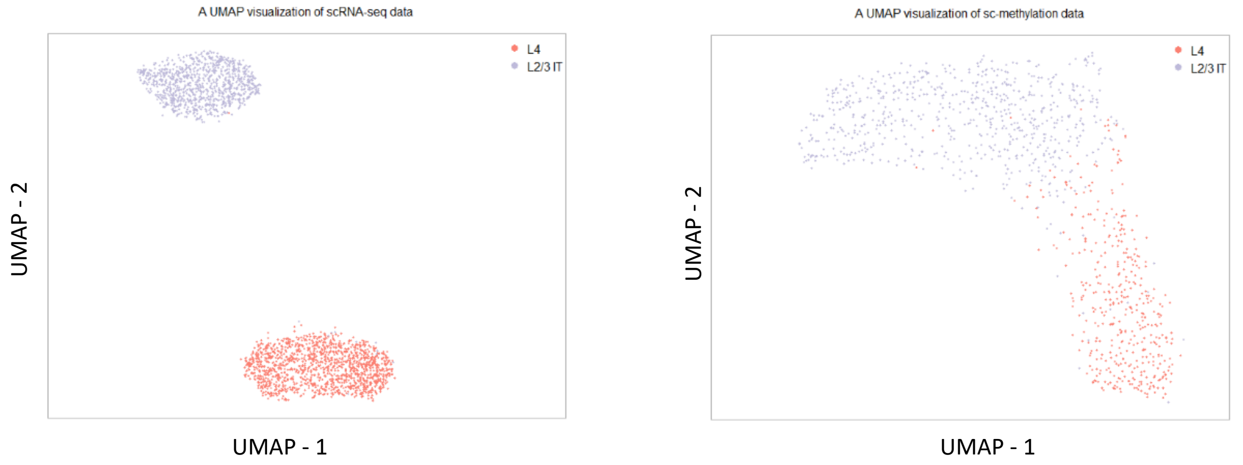

Figure R.3: UMAP visualization of source data (left) and target data (right) in example 3.

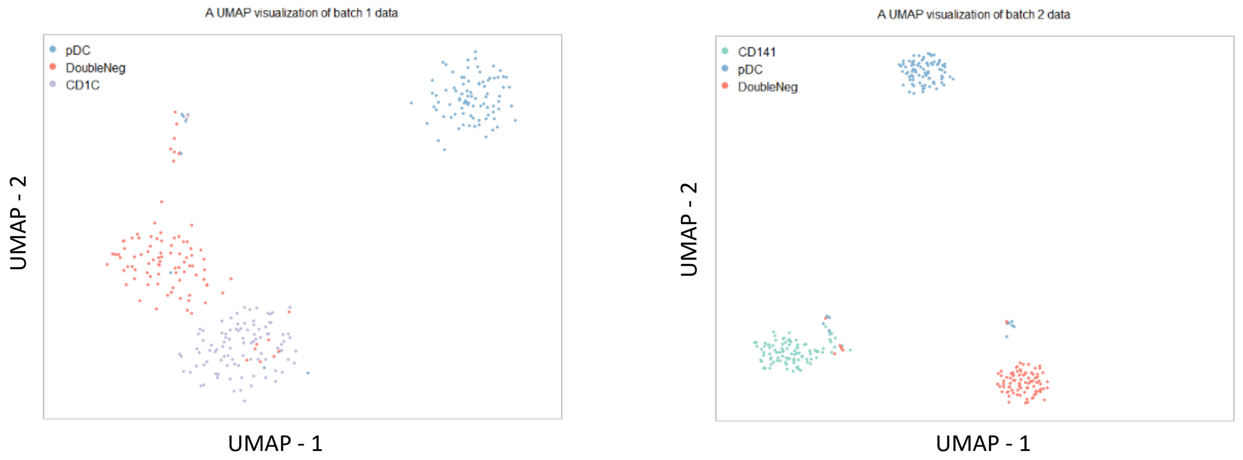

Figure R.4: UMAP visualization of source data (left) and target data (right) in example 4.

of one modality, which facilitates denoising and helps distinguish the cell types in the other modality. This is demonstrated by the superiority of *couple*CoC+ over CoC (which does not transfer knowledge between two datasets) in real data examples 1-3 in the manuscript. In practice, we would choose the modality with less noise and better power in separating the cell types as the source data, and the other modality as the target data.

4. For the major figures (the heatmaps in Figure 2-4), the horizontal labels and the vertical labels

overlap and can be confusing. My understanding is the horizontal labels are clustering of features, while the vertical labels are for cells - if there is no matched cell cluster in the target data, the labels overlap.

Response:

Thank you for your comment. We apologize for not making the labels clear.

In the revised manuscript, to distinguish cell cluster and feature cluster and to avoid the overlap of labels, we use "clu m" to represent the matched cell clusters in source data and target data, "clu s" to represent the cell clusters that are unique to the source data, and "clu t" to represent the cell clusters that are unique to the target data. We use "clu" to represent the feature clusters. We changed all these notations for the heatmaps in Figures 2-5 and added the descriptions in each figure caption.

Thank you again for your meticulousness.

5. For integrating scRNA-seq with scATAC-seq, the authors use the "gene activity" calculated from the ATAC reads as the link features. It has been shown that the accessibility in promoter region is not always a good proxy for pseudo-gene expression. Would this be an issue? Any comments on this?

Response:

Thank you for your comment.

Yes, promoter accessibility is not always a good proxy for pseudo-gene expression. For example, during embryonic development, a large proportion of the promoters tend to be accessible. This is exactly the reason that we use gene activity score, instead of promoter accessibility. The example that we presented in the manuscript uses gene activity score. Gene activity score summarizes the accessibility of the regions around the gene (including both promoter and other regions nearby), where the weights of the regions decay with the distance to the gene. We added the clarification in the *Materials and methods* section: "Promoter accessibility may also be used to link scATAC-seq data with scRNA-seq when gene activity score is not available. Promoter accessibility may have less power in separating the cell types compared with gene activity score, because gene activity score incorporates more regions nearby the gene." To utilize more information in scATAC-seq data, we also include other more distal regions that are unlinked to the genes as the unlinked features in *couple*CoC+.

Have all data underlying the figures and results presented in the manuscript been provided? Large-scale datasets should be made available via a public repository as described in the *PLOS Computational Biology* data availability policy, and numerical data that underlies graphs or summary statistics should be provided in spreadsheet form as supporting information.

NO: Source data and analytical scripts are not available.

Response:

Thank you for your reminder.

The processed data (including simulation data) for the input of *couple*CoC+ are available at `https://github.com/cuhklinlab/coupleCoC_plus/tree/main/data`. **The followings are the information for the original unprocessed datasets. The mouse cortex scRNA-seq data in example 1 and example 3 are available at NCBI Gene Expression Omnibus (GEO) under accession GSE115746. The mouse cortex scATAC-seq data in example 1 were downloaded from** `https://atlas.gs.washington.edu/mouse-atac/data/`. **The mouse and human scRNA-seq data in example 2 are available at** `https://panglaodb.se/view_data.php?sra=SRA832392&srs=SRS4237518` **and** `https://panglaodb.se/view_data.php?sra=SRA878024&srs=SRS4660846`**, respectively. The mouse cortex sc-methylation data in example 3 are available at GEO under accession GSE97179. The human blood dendritic cells scRNA-seq data in example 4 are available at GEO under accession GSE94820.**

The source code for the analysis is available at `https://github.com/cuhklinlab/coupleCoC_plus` **and in "vignette.md" at github (**`https://github.com/cuhklinlab/coupleCoC_plus/blob/main/vignettes/vignette.md`**).**

In the revised manuscript, we added these details in the section: *Data and code availability.*

Reviewer #2

In this paper, authors proposed *couple*CoC+, which is based on the information-theoretic co-clustering transfer learning framework for the integrative analysis of single-cell genomic data. The goal of *couple*CoC+ is to utilize one dataset, the source data (S), to facilitate the analysis of another dataset, the target data. Depending on whether the features are linked with the source data or not, the target data can be partitioned into two parts, data T with the linked features, and data U with the unlinked features. The authors introduced a well-studied concept in information to solve an important computational biology problem. I have following questions.
Major comments:

1. In this article, authors use the real examples to demonstrate that the proposed method has better performance in clustering target data with source data at single-cell level. However, the simulation studies are missing to show that under which cases, *couple*CoC+ is better and under which cases, *couple*CoC+ does not provide gains.

Response:

Thank you for your constructive comment.

In the revised manuscript, we added a section: *Simulation studies* with 6 settings to test the performance of *couple*CoC+. Our observation is that when the unlinked features (data U) provide no information in separating the cell types (setting 5), *couple*CoC+ is slightly worse than *couple*CoC; when source data S is much noisier than target data T, *couple*CoC+ and *couple*CoC do not perform well, compared with methods that do not transfer knowledge from the source data. The followings are the details for the simulation settings and the results.

We followed the simulation setup given in Lin et al. (2019) with some modifications specific to our framework. The details for generating data T, S and U are given in Supplementary Text. We set the numbers of cell types in both target data and source data as $N_{\mathbf{T}} = N_{\mathbf{S}} = 2$, set the numbers of cells as $n_{\mathbf{T}} = n_{\mathbf{S}} = 100$, set the proportion of each cell type as 0.5 and set the number of features as $q = q_0 = 1000$. We varied the differential degree (w) across clusters, the standard deviations ($\sigma_{\mathbf{S}}$ and $\sigma_{\mathbf{T}}$, corresponding to source data and target data, respectively) of the generative distribution, and the shift between two means (d) of the generative distributions for the two cell types in data U (i.e., unlinked features in target data). Larger w leads to better separation of the cell clusters in data S and data T, larger $\sigma_{\mathbf{S}}$ or $\sigma_{\mathbf{T}}$ leads to higher noise level in data S or data T, and larger d leads to better separation of the cell clusters in data U. We considered six different simulation settings, by varying the parameters w, $\sigma_{\mathbf{S}}$, $\sigma_{\mathbf{T}}$ and d. We compared *couple*CoC+ with *couple*CoC and CoC.

Table R.1: The simulation results for clustering the target data in 30 independent runs are summarized. Note that the capital letters in the brackets represent the input data matrices for the corresponding methods: S represents source data, T and U represent the sub-matrices for the linked and unlinked features in target data, respectively. *couple*CoC+ and *couple*CoC utilize both the source data and the target data as input, and they produce clustering results of the cells in source data and target data simultaneously; CoC is implemented on only target data, and it produces clustering results of the cells in target data. We then summarize the clustering results by calculating ARI and NMI for target data.

Clustering methods	Setting 1 $w=0.67, d=2$ $\sigma_S=\sigma_T=1.4$		Setting 2 $w=0.67, d=2$ $\sigma_S=\sigma_T=1.7$		Setting 3 $w=0.64, d=2$ $\sigma_S=\sigma_T=1.4$		Setting 4 $w=0.64, d=1$ $\sigma_S=\sigma_T=1.4$		Setting 5 $w=0.64, d=0$ $\sigma_S=\sigma_T=1.4$		Setting 6 $w=0.64, d=2$ $\sigma_S=2.0, \sigma_T=1.4$	
	ARI	NMI	ARI	NMI	ARI	NMI	ARI	NMI	ARI	NMI	ARI	NMI
*couple*CoC+(S+T+U)	0.935	0.905	0.855	0.802	0.834	0.805	0.816	0.711	0.780	0.699	0.637	0.601
*couple*CoC(S+T)	0.909	0.874	0.824	0.750	0.800	0.746	0.800	0.746	0.800	0.746	0.611	0.555
CoC(T+U)	0.826	0.781	0.816	0.774	0.810	0.766	0.567	0.522	0.002	0.009	0.812	0.765

Table R.1 presents the simulation results for the target data. In settings 1-3, we fixed $d=2$, and we varied w, $\sigma_\mathbf{S}$ and $\sigma_\mathbf{T}$. Compared with setting 1 ($w=0.67, \sigma_\mathbf{S}=\sigma_\mathbf{T}=1.4$), setting 2 has higher noise ($\sigma_\mathbf{S}=\sigma_\mathbf{T}=1.7$), and setting 3 has lower differential ability across the cell clusters ($w=0.64$) in data T and data S. Compared with setting 3 ($d=2$), the unlinked features (data U) in target data have less power in separating the cell types in setting 4 ($d=1$). In these four settings, *couple*CoC+ performs better than *couple*CoC, because data U provide information for separating the cell types and *couple*CoC does not utilize the information in data U. In setting 4, where d is smaller, i.e. data U have less power in separating the cell types, the margin between *couple*CoC+ and *couple*CoC becomes smaller. Both *couple*CoC+ and *couple*CoC have better clustering performance than CoC, due to the transfer of knowledge from source data to clustering target data. When data U contain no information in separating the cell types (setting 5, $d=0$), the performance of *couple*CoC+ is slightly worse than *couple*CoC. CoC does not work well in setting 5 because it is affected by data U and it does not transfer knowledge from the source data. When source data S have higher noise (setting 6, $\sigma_\mathbf{S}=2.0$), the performance of *couple*CoC+ and *couple*CoC drops and they become inferior to CoC. *couple*CoC+ is slightly better than *couple*CoC in setting 6, because it incorporates the information in data U.

2. In Table 1, it is not clear whether the summarized results are calculated by applying ARI and NMI to target or source data, or both.

Response:

Thank you for your comment. We apologize for not making it clear in the previous version of the manuscript.

For integrative analysis methods (*couple*CoC+, *couple*CoC, Seurat, LIGER, scACE) that utilize both the source data and the target data as input, they produce clustering results of the cells in source data and target data simultaneously. We then summarize the clustering results by calculating ARI and NMI for source data and target data separately. For the remaining methods that are implemented on only one dataset, they produce clustering results of the cells in source data or target data independently. We then summarize the clustering results by calculating ARI and NMI for source data and target data separately. We have clarified this in the captions of Tables 1-2 in the revised manuscript.

3. Authors demonstrate *couple*CoC+ using three real examples. I am wondering any biological interpretations to the clustering results. It would be helpful to show the biology of findings.

Response:

Thank you for your constructive comment.

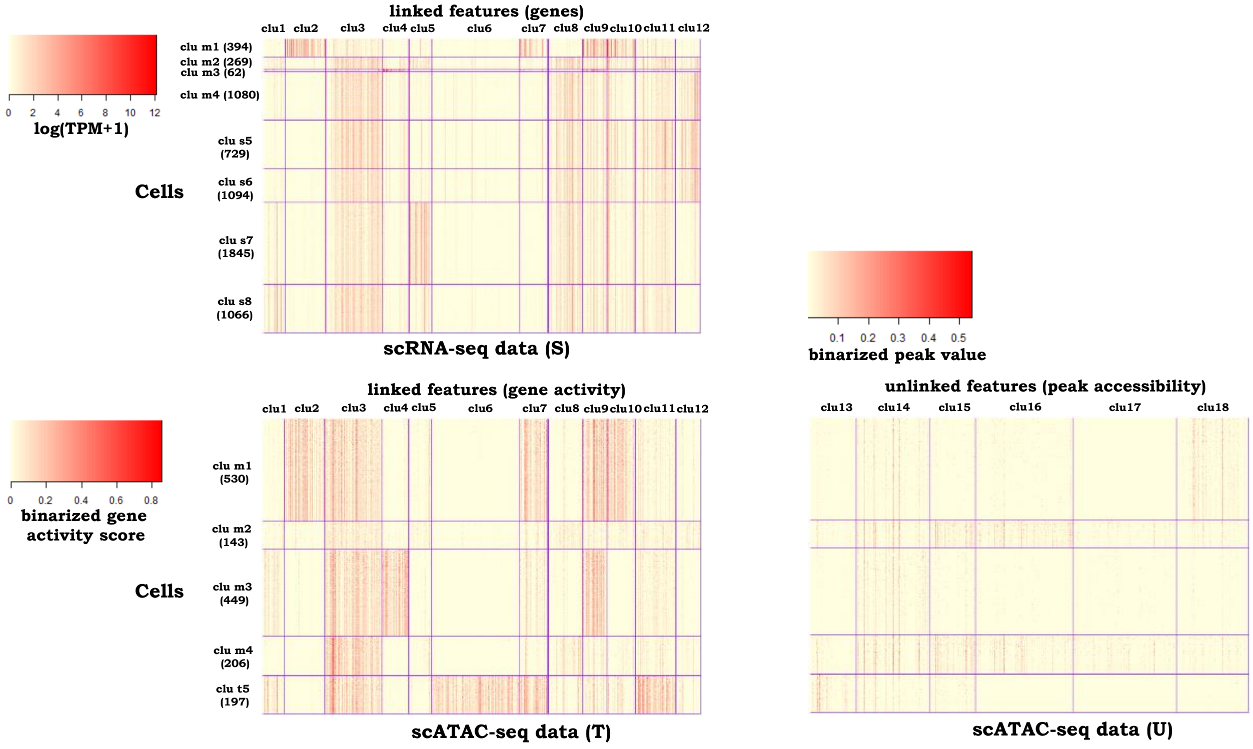

Figure R.5: Heatmaps of the clustering results by *couple*CoC+ for example 1. “clu m” represents the matched cell cluster across the source data and the target data. “clu s” and “clu t” represent the cell clusters that are unique to the source data and the target data, respectively.

The genes that are clustered together by *couple*CoC+ tend to be enriched for functional annotation terms closely related to the cell clusters in which they are active. More specifically, we investigated the features that are clustered together by *couple*CoC+ in example 1 (Figure R.5). Feature cluster "clu4" is specific to cell cluster "clu m3" in scRNA-seq and scATAC-seq data, which are mostly oligodendrocyte cells; and feature cluster "clu6" is specific to "clu t5" in scATAC-seq data, which are mostly microglia cells. We performed functional annotation enrichment analysis using DAVID (Huang et al., 2009a,b). The genes in feature cluster "clu4" (59 genes in total) are highly enriched for the terms related to myelin (more comprehensive list in Table R.2). The top three terms and their Bonferroni corrected *p*-values are ("myelin sheath", 1.44×10^{-11}), ("myelination", 1.12×10^{-7}) and ("structural constituent of myelin sheath", 2.51×10^{-5}), respectively. By creating myelin sheath, oligodendrocytes provide support and insulation to axons in the central nervous system. The genes in feature cluster "clu6" (198 genes in total) are highly enriched for the terms related to the immune system (more comprehensive list in Table R.3). The top two terms and their Bonferroni corrected *p*-values are ("immunity", 8.27×10^{-22}) and ("immune system process", 3.00×10^{-19}), respectively. Microglia represents a specialized population of macrophages-like cells in the central nervous system (CNS) considered immune sentinels that are capable of orchestrating a potent inflammatory response (Sara et al., 2018).

We added these biological interpretations in example 1 in the revised manuscript and added these two tables in the revised supporting information file *S3 Supplementary Tables* (Table S.2. and Table S.3.).

Table R.2: Enriched functional annotation terms for gene list in the "clu 4" of linked genes in example 1 using DAVID tools. The top 10 terms are shown here.

Category	Term	Count	%	Bonferroni P-value
GOTERM_CC_DIRECT	myelin sheath	13	22.03	1.44E-11
GOTERM_BP_DIRECT	myelination	8	13.56	1.12E-07
GOTERM_MF_DIRECT	structural constituent of myelin sheath	4	6.78	2.51E-05
UP_KEYWORDS	Glycoprotein	24	40.68	0.00179
UP_KEYWORDS	Disulfide bond	21	35.59	0.00358
GOTERM_BP_DIRECT	peripheral nervous system myelin maintenance	3	5.08	0.0732
UP_SEQ_FEATURE	glycosylation site:N-linked (GlcNAc...)	23	38.98	0.0501
UP_SEQ_FEATURE	topological domain:Extracellular	16	27.12	0.302
GOTERM_CC_DIRECT	extracellular exosome	17	28.81	0.172
UP_SEQ_FEATURE	lipid moiety-binding region:S-palmitoyl cysteine	5	8.47	0.337

Table R.3: Enriched functional annotation terms for gene list in the "clu 6" of linked genes in example 1 using DAVID tools. The top 10 terms are shown here.

Category	Term	Count	%	Bonferroni P-value
UP_KEYWORDS	Immunity	35	17.68	8.27E-22
GOTERM_BP_DIRECT	immune system process	34	17.17	3.00E-19
UP_KEYWORDS	Disulfide bond	81	40.91	5.02E-19
GOTERM_BP_DIRECT	inflammatory response	29	14.65	2.81E-15
UP_SEQ_FEATURE	topological domain:Extracellular	69	34.85	2.15E-15
UP_KEYWORDS	Glycoprotein	84	42.42	1.79E-15
UP_KEYWORDS	Innate immunity	23	11.62	2.10E-14
UP_SEQ_FEATURE	topological domain:Cytoplasmic	75	37.88	3.54E-13
UP_SEQ_FEATURE	disulfide bond	69	34.85	7.79E-13
GOTERM_CC_DIRECT	membrane	121	61.11	1.72E-12

4. As batch effects are major obstacles to perform integrative analysis using single-cell data, I am wondering if *couple*CoC+ is capable of integrating datasets from different batches. It would be helpful to discuss it.

Response:

Thank you for your constructive comment.

We added a fourth real data example to examine *couple*CoC+ by integrative clustering of human blood dendritic cell (DC) scRNA-seq data from two batches (Villani et al., 2017). Each batch consists of 96 CD141 DC, 96 CD1C DC, 96 plasmacytoid DC (pDC) and 96 double negative cells. The data were generated from the Smart-Seq2 platform and they were used in a recent benchmark study (Tran et al., 2020). We processed the data similar to Tran et al. (2020), where CD141 DC in batch 1 and CD1C DC in batch 2 were removed. So, both batches share pDC and double negative cells, and each batch has one unshared cell type (CD1C and CD141 respectively) that are biologically similar. We chose batch 1 as the source data and chose batch 2 that is sparser as the target data (The proportions of zero entries in batch 1 and batch 2 are 32.65% and 42.89%, respectively.). We set the number of matched clusters N_{sub} as 2, because the values of the objective function $g(N_{\text{sub}})$ for choosing N_{sub} are smallest when $N_{\text{sub}} = 2$: 8.66×10^{-4} when $N_{\text{sub}} = 1$, 6.37×10^{-4} when $N_{\text{sub}} = 2$, and 1.08×10^{-3} when $N_{\text{sub}} = 3$.

All five integrative methods, except *sc*ACE, have good clustering performance for scRNA-seq data in batch 1 (source data)(Table R.4). *sc*ACE also fails to cluster scRNA-seq data in batch 2 (target data). Because no unlinked features are included in target data, *couple*CoC+ and *couple*CoC have similar clustering performance for the data from two batches, and they have competitive performance compared with the other methods (Table R.4). Figure R.6 is the corresponding heatmap after clus-

Table R.4: The results of clustering the cells in target data and source data in the fourth example.

	*couple*CoC+	*couple*CoC	Seurat	LIGER	scACE
Target data (batch 2)					
ARI	0.837	0.838	0.777	0.729	0.496
NMI	0.804	0.800	0.728	0.667	0.479
Source data (batch 1)					
ARI	0.858	0.858	0.830	0.800	0.489
NMI	0.819	0.819	0.789	0.731	0.493

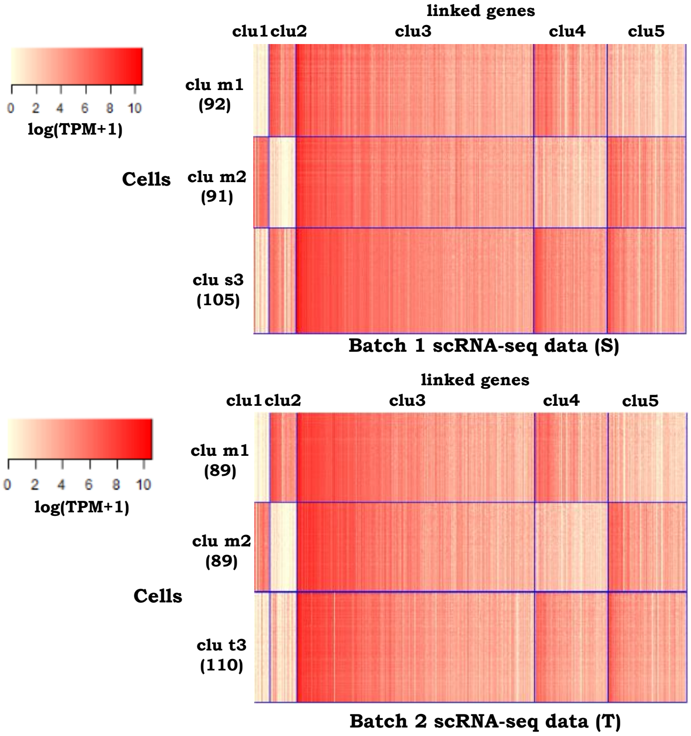

Figure R.6: Heatmaps of the clustering results by *couple*CoC+ for example 4. "clu m" represents the matched cell cluster across the source data and the target data. "clu s" and "clu t" represent the cell clusters that are unique to the source data and the target data, respectively. For better visualization, we randomly averaged every 15 cells within the same cell cluster to generate pseudocells for every heatmap.

tering by *couple*CoC+. *couple*CoC+ clearly clusters similar cells and features, and it accurately found the two matched clusters (the shared pDC and double negative cells) across the two batches. In addition, the expression patterns for the matched clusters tend to be consistent in the two batches. The cell types unshared by the two batches, including CD1C DC and CD141 DC, are represented by "clu s3" and "clu t3", respectively. They have high similarity with each other, and they are only distinguished by feature cluster "clu2".

References

Huang, D. W., Sherman, B. T., and Lempicki, R. A. (2009a). Bioinformatics enrichment tools: paths toward the comprehensive functional analysis of large gene lists. *Nulceic Acids Res.*, 37(1):1–13.

Huang, D. W., Sherman, B. T., and Lempicki, R. A. (2009b). Systematic and integrative analysis of large gene lists using david bioinformatics resources. *Nature Protoc.*, 4(1):44–57.

Lin, Z. X., Zamanighomi, M., Daley, T., Ma, S., and Wong, W. H. (2019). Model-based approach to the joint analysis of single-cell data on chromatin accessibility and gene expression. *Stat. Sci.*

Sara, B., Itzia, J. F., Agnes, P., and the others (2018). Microglia in neurological diseases: A road map to brain-disease dependent-inflammatory response. *Front. Cell. Neurosci.*

Tran, H., Ang, K., Chevrier, M., and the others (2020). A benchmark of batch-effect correction methods for single-cell rna sequencing data. *Genome Biology*, 21(12).

Villani, A.-C., Satija, R., Reynolds, G., and the others (2017). Single-cell rna-seq reveals new types of human blood dendritic cells, monocytes, and progenitors. *Science*, 356(6335).
